# Engineering student wellbeing in hybrid learning environments post-COVID-19: a call for targeted mental health interventions

**DOI:** 10.3389/fpsyg.2025.1694258

**Published:** 2025-12-03

**Authors:** Maria Cristina Medrano Firmante

**Affiliations:** Counseling and Psychological Services, De La Salle University, Manila, Philippines

**Keywords:** engineering students, post-pandemic, hybrid learning, adjustment, mental health intervention

## Introduction

The COVID-19 pandemic has dramatically transformed the higher education landscape. To maintain academic continuity while minimizing health hazards, universities globally changed their modes of learning from traditional to online studies. Post this, numerous institutions have turned to hybrid learning models- a mix of online and sparse face-to-face instruction. Though this has facilitated flexibility and ease of access, it has also posed a fresh slate of problems, especially for students pursuing courses in engineering.

There are unique requirements for engineering education, such as rigorous coursework, high academic standards, and a curriculum that frequently emphasizes experiential, cooperative learning. Before the pandemic, engineering students were already recognized as a high-risk demographic for excessive stress and burnout. These factors were only exacerbated by the move to hybrid learning. Based on studies, engineering students tend to fall more into emotional exhaustion, digital boredom, incoherent peer interaction, and a very ambiguous personal and academic life (Firmante, 2024; Khorshid et al., 2024; Sharma et al., 2024). Filipino first-year engineering students found it difficult to cope with hybrid environments and experienced psychological stress and social isolation (Firmante, 2024). Additionally, a narrative synthesis of stories revealed that engineering students' lower psychological wellbeing is often related to the demand of the hybrid learning (Firmante, 2025). To provide a conceptual framework for understanding these outcomes, one can examine the stressors of hybrid learning through the lens of established models of psychological distress and coping. For instance, the stress and coping theory of Lazarus (2006) and Lazarus and Folkman (1984) is an example of how the students recognize and react to the demands of hybrid learning while the diathesis-stress model (Salomon and Jin, 2013) explains the reason why some students might be more prone to stress-related outcomes. Moreover, the Academic Psychological Distress model and the scales of college adjustment (Khatri et al., 2024; O'Donnell et al., 2018; Scandurra et al., 2024, 2025) are examples of student-centered frameworks that not only stress the interplay between the academic and social dimensions of hybrid learning that might cause tension but also recognize the impact of these dimensions on the student's holistic health. To address these issues, researchers, medical professionals, and educators are looking for solutions that will lessen the risks to mental health in this innovative learning setting.

According to Kalamatianos et al. (2025), an interdisciplinary counseling intervention improved engineering students' coping skills and stress levels. Jensen (2021) argues this is the most opportune moment to instill a lasting wellness culture in engineering education. Expanding telecounseling services, sponsoring virtual non-academic extracurriculars, and incorporating mental health education into the curricula are some of the most effective approaches. Digital training modules stabilize students' mental health regarding mindfulness, stress management, and wellness techniques.

The post-pandemic academic period demands that public health experts, university leaders, and engineering teachers acknowledge student mental health as essential to educational quality and fairness. Our educational frameworks require simultaneous advancement with mental health support systems. Establishing a consistent approach for psychological wellness would help academic institutions improve digital telehealth services in hybrid settings. Encouraging engineering student' wellbeing is an investment in the future of science, technology, and innovation as well as an educational issue.

## The psychological toll of hybrid learning on engineering students

According to recent studies, hybrid learning negatively affects students' mental wellbeing in countries with high academic standards. In the Philippines, for instance, first-year engineering students reported high psychological stress levels and learning difficulties in an integrated learning setting. Among the main concerns, students cited social isolation and a lack of academic support (Firmante, 2024). Engineering students in hybrid formats also experienced high level of stress, digital burnout, and emotional weariness (Khorshid et al., 2024; Sharma et al., 2024).

For some, the asynchronous nature of hybrid courses created uncertainty and lack of motivation while frequent switching between online and in-person modes created confusion and logistical burden. The shift from traditional, face-to-face experiential learning has also created gaps in academic continuity and peer interaction- crucial to effective engineering education and a sense of academic belonging. A 2025 narrative synthesis by Firmante reinforces these findings, concluding that hybrid learning environments are closely associated with reduced psychological wellbeing among engineering students. The synthesis highlighted that the absence of organized emotional and academic support systems particularly during the crucial academic transitions like for the first-year students has intensified students' adjustment difficulties. The study specifically observed that students from under-resourced or marginalized backgrounds were more susceptible to these effects, underscoring the need for equity centered interventions. Significantly, these results indicate that the mental impacts of hybrid learning are not temporary issues confined to the pandemic period. Rather, they indicate systemic shortcomings in how educational institutions equip and assist students in adjusting to contemporary, adaptable learning settings. Lacking sufficient mental health resources and culturally relevant programs, hybrid education may exacerbate existing disparities and negatively affect student outcomes. Theoretically, the difficulties encountered in hybrid learning mentioned earlier can be linked to the stress and coping theory as well as the academic psychological distress model. These connections highlight that the stress and coping model and academic psychological distress model predict the occurrence of stress and coping problems with hybrid learning. This also brings out the urgent need for targeted interventions to prevent the onset of negative mental health outcomes.

## Interventions and recommendations for mental health support

The necessity for proactive, scalable, and evidence-based mental health programs and initiatives is emphasized by educators, administrators, and mental and healthcare specialists in light of the reported mental health issues that engineering students face in hybrid learning contexts. Integrating mental health support as a fundamental pillar of hybrid engineering education is critical to fostering sustainable academic success and wellbeing.

## Interdisciplinary counseling programs

In a 5-week blended counselor program designed with Greek undergraduate engineering students, Kalamatianos et al. (2025) integrated cognitive behavioral therapy and positive psychology. Researchers noted that while anxiety was higher or unchanging among students, stress and depression were notably lower than in an untreated control group. This intervention also demonstrates the benefits of an integrated model that combines psychological counseling, academic advising, and peer support. Integrated programs recognize the compound effects of stressors on students and aim to provide an alternative to fragmented mental health systems and services, particularly those that rely on student services to reach counseling centers within academic departments and colleges. Integrated psychological and academic support systems models show the connection between student performance and psychological wellbeing, rather than as separate issues (Kalamatianos et al., 2025). In hybrid environments, when students are dealing with emotional stressors brought on by social isolation and digital weariness in addition to scholastic difficulties, integrated models might also be helpful (Firmante, 2025).

## Embedding mental health education into the curriculum

In addition to counseling, the integration of mental health education into engineering curricula represents a potential proactive approach to fostering resilience at some time of longevity. Jensen (2021) notes that the post-pandemic context has provided an important opportunity for engineering programs to implement a culture of wellness. Jensen suggests that this culture of wellness can be accomplished via mental health literacy, as opposed to technical literacy, in engineering programs. Jensen also suggests digital or partially digital modules on wellness, mindfulness, emotion management, stress management, and resilience building could fit into first-year seminars or orientation. Capacity-building strategies like Jensen's have been remarked by other authors as providing students with coping tools that will assist in navigating academic requirements and personal stressors, and if delivered in an early manner can create resilience that minimizes possible burnout and disengagement (Jensen, 2021). Raising awareness of wellness early in the experience appears to be critical, as first year students experiencing hybrid learning may found the transition from in school learning to online/hybrid modalities difficult (Firmante, 2024). Moreover, in order to ensure student relevance of the content and practice what they learn about co-creation and responsiveness, students should have a role in creating wellness modules similar to the one described above. These might strategies that develop healthy digital boundaries; place time management strategies in the hybrid/online context; and involve peer to peer collaboration that has built in degrees of separation (Sharma et al., 2024). Notably, involvement to create “buy in” utilizes the principles of strength-based approaches to ensure student empowerment in the space of wellness, building habits on student ownership of mental health.

## Telecounseling and virtual community building

Telecounseling has emerged as a vital modality to extend mental health services beyond traditional campus settings, particularly within hybrid and remote learning frameworks. There has been an increase in attendance in universities where telecounseling services have formed among students who do not visit due to stigma, inaccessibility of visits, time constraints, or social anxiety (Khorshid et al., 2024). The flexibility, privacy, and accessibility offered by telecounseling align well with hybrid education models, ensuring continuous psychological support regardless of students' physical location. Among Filipino engineering students, telecounseling promotes the formation of virtual communities that help counter the prevalent sense of social isolation along with virtual wellness workshops, mental health awareness campaigns, and peer-led student support groups (Firmante, 2025). In addition, universities should offer mental health first aid training for faculty members and staff, and include routine wellbeing check-ins as part of academic interactions. Faculty are usually the frontline observers of student distress- sometimes, they are the first to identify a student in distress and can provide timely referrals to appropriate, supportive services. Embedding mental health advocacy into the normal course of academic life allows for the normalization of conversations around wellbeing and decreases stigma. It is also a way to support student wellness and to communicate the priorities of wellbeing alongside academic success (Jensen, 2021; Kalamatianos et al., 2025).

## Discussion

The post-pandemic academic setting offers us an opportunity to, most importantly, reshape student support systems, particularly in areas such as engineering. The hybrid learning has not only revealed but also made the unduly stressed and burned out students more vulnerable psychologically in and among students. It is imperative that we change the perspective of the whole educational institution—the acknowledgment and the support of the mental health of engineering students is not a social intervention but rather an investment in the quality assurance of higher education and the global engineering workforce's sustainable success that will last for a long time. This will only be possible when a system-level infrastructure is built that goes beyond occasional crisis management. Among the changes to take place are establishing a continuous loop of care by regular wellbeing assessments, providing appropriate broadband and telehealth resources, and teaching mental health literacy in classroom practices. This change must be accompanied by equitable methods and work specifically with the most vulnerable and least resourced students who suffer from the combined effects of digital exclusion and financial hardship. Moreover, institutions focusing on psychological wellness will be able to reap the benefits in the form of better academic performance, lower dropout rates, and higher postgraduate aspirations. It is through the development of engineering students' psychological resilience that the return on investment in the classroom is realized as well as in the innovative and problem-solving capabilities they bring to the workforce. Those institutions that decide to lead in this matter will not only uplift their students' welfare but will also, in a way, determine the future of higher education as a progressive, adaptable, and even humanistic education.

## Conclusion

The academic environment that has evolved post-pandemic, provides a chance to, above all, re-construct the student support systems, especially in the case of the engineering field. The mixed learning mode has uncovered the problem and made the already troubled students suffer more in terms of their mental health. The change in attitude of the whole educational institution is very important—the recognition and the support of the mental health of engineering students is not a social intervention but an investment in the quality assurance of higher education and the global engineering workforce's sustainable success that will last for a long time. This will only happen if the foundation for a system-level infrastructure that surpasses the ever-present crisis management is constructed. To this end establishing proper and continuous monitoring of the students' wellbeing, offering appropriate broadband and telehealth resources, and integrating mental health literacy into classroom practices are among the changes to be made. This transition should be accompanied by fair practices and should specifically target the most at-risk and least well-off students who are suffering from a lack of access to digital technology and financial hardship. Institutions that prioritize mental health will not only be able to enjoy better students' academic performance, lower dropout rates, and higher postgraduate aspirations but also reap the benefits of such investments in future professionals. It is the psychological resilience of engineering students that makes the return on investment in the classroom and the innovative and problem-solving capabilities they bring to the workforce. Those institutions that choose to be at the forefront in this matter will not only enhance the welfare of their students but in some way also shape the future of hi-tech industries.
